# The Afrotropical *Miomantis caffra* Saussure 1871 and *Miomantis paykullii* Stal 1871: first records of alien mantid species in Portugal and Europe, with an updated checklist of Mantodea in Portugal (Insecta: Mantodea)

**DOI:** 10.3897/BDJ.2.e4117

**Published:** 2014-11-12

**Authors:** Eduardo Marabuto

**Affiliations:** † Computational Biology and Population Genomics Group, Centro de Biologia Ambiental, Faculdade de Ciências, Universidade de Lisboa, Campo Grande, 1749-016, Lisboa, Portugal; ‡ Centro de Estudos do Ambiente e do Mar, Departamento de Biologia, Universidade de Aveiro, Portugal, Aveiro, Portugal

**Keywords:** New records, Western Europe, Biological invasion, biotic homogenisation, mantis, key to species

## Abstract

The recent growing interest on the Mantodea fauna of southern Europe and Portugal in particular, has enabled the discovery of two geographically separated populations of hitherto unknown species in Europe. Analysis of specimens shows that they belong to two Afrotropical mantids: *Miomantis caffra* Saussure, 1871 and *Miomantis paykullii* Stal, 1871, thus raising the number of known species in Europe to 39 and in Portugal to 11.

While these are remarkable findings, they also represent the first alien mantis species recorded from this continent. As yet, these species appear to be confined to artificial humanised gardened areas but call for more attention to the problem of biological invasions and the need for better bio-security measures for the conservation of natural ecosystems.

In the absence of recent revisionary work on the Mantodea of Portugal and given the need to provide an accessible identification tool, both a checklist and a key to species are provided for all species in the country.

## Introduction

Human impact on biological diversity can occur at different levels and magnitudes. The effect on ecosystems can be direct, by their removal or alteration, or manifest themselves indirectly such as through climate change or the translocation of species between different biogeographic regions. Generally, due to an incomplete knowledge of ecosystems by researchers and policy-makers, many introduction cases may be overlooked, even more rarely tracked down precisely as they happen. Therefore, their effect has mostly been under-evaluated. Competition with natives for resources and other environmental constraints limits the successful establishment of most alien species (Elton 1958), and if they do establish there is a chance the effect is not visible right away, especially if crops are not involved.

The term "alien species" generally includes those naturally exogenous to a given habitat, ecosystem or biogeographical area which have established themselves outside their natural range either unaided or with human assistance. Several paths and vectors for introduction of such species have been identified (e.g. Hulme et al. 2008). These include deliberate or unintentional ways, the prevalence of which differs according to the biological group. Introductions, particularly if mediated by man are fundamentally different from natural range expansions both quantitatively and qualitatively. In contrast to natural dispersal, which usually involves highly mobile organisms with a broad ecological plasticity, these may include species with reduced ability to overcome important physiological barriers (e. g. slugs or amphibians arriving on oceanic islands) or have distant origins (e. g. the introduction of reindeer in sub-Antarctic islands) (Wilson et al. 2009).

In Europe there are over 1500 established alien arthropod species, of which 1390 are insects (Roques et al. 2009). According to the DAISIE database, the largest joint project to identify alien species in the European territory (DAISIE 2009 and www.europe-aliens.org), the major pathway of entrance and establishment of species in the territory is unintentional direct introduction (1341, 86%). Of these, introduction with horticultural and ornamental items accounts to 468 species (29%). 'Only' 218 species have been supposedly intentionally introduced into the European territory, mostly as biological control agents (Rabitsch 2010). It is estimated that 24.2% have an economic impact (Vilà et al. 2010) and that the majority of aliens settle preferably in humanised, altered habitats (65%, 1040 species), i.-e. that depart from the natural local assemblages (Lopez-Vaamonde et al. 2010). Parks and gardens (31.4%, 500 species), houses and built areas (31%, 493 species) and then agricultural land (29.7% or 472 species) are the preferred allochthonous habitats.

While mantises have so far not been reported as alien species in Europe, the worldwide scenario is quite different. From as early as 1899, *Mantis religiosa* L. was introduced into USA and Canada with nursery plants (McLeod 1962, Cannings 2007), alongside the Oriental *Tenodera sinensis* (Helfer 1987, Hurd 1999). *Iris oratoria* (Linnaeus, 1758), another Euro-Mediterranean species arrived in California during the thirties (Maxwell and Eitan 1998) from where it seems to be spreading. Finally, the south African *Miomantis caffra* has been established in New Zealand for almost forty years (Ramsay 1984).

The European Mantodea species list (Heller and Bohn 2013) includes around 38 species belonging to four distinct families (Amorphoscelidae, Empusidae, Mantidae and Tarachodidae). Mantidae is by far the richest family with 30 species (Battiston et al. 2010) and all but the genus *Ameles* are of straightforward identification. As for now, *Ameles* is still under scientific scrutinity as authors debate the status of several taxa (Agabiti et al. 2010, Battiston and Fontana 2005, Battiston et al. 2010, Obertegger and Agabiti 2012, Wieland et al. 2014). In the meantime, Fauna Europeaea (Heller and Bohn 2013) seems not to be up to date with recent findings and the number of *Ameles* spp. in the region may be around eight (*A. assoi*, *A. decolor*, *A. fasciipennis*, *A. heldreichi*, *A. insularis*, *A. paradecolor*, *A. picteti* and *A. spallanzania*).

Irrespective of the criteria for the definition of species and biogeographical structure, the most up-to-date and synthetic overall work on Euro-Mediterranean Mantodea, Battiston et al. 2010, gave 127 species from a wide area from the Canary Islands to the Caucasus, all Europe, North Africa and Mediterranean islands. Diverse and heterogeneous landscapes, different land-uses, rugged topography and the character of refugia-expansion cycles and contact zones during and after past glaciations allow for an interesting biological diversity in this crossroad between three different continents. However, there is a great south-north disparity and because of their typical thermophilous profile, the Mantodea attain highest diversity in warmer areas of the southern Mediterranean. On the other hand, the aridity and lack of many suitable habitats renders the Sahara desert unsuitable and an effective barrier to interchangeability of biota with the Afrotropical region. The only exception is the corridor represented by the Nile river valley, promoting the contact and migration of species from deep in east Africa to the Mediterranean. Therefore, Egypt has the greatest diversity of mantis species in the region, some of which are clearly of Afrotropical origin like the genera *Heterochaeta* and *Miomantis*.

In the summer of 2014, unexpected findings of two mantis species in Portugal triggered a revision of the species occurring in the country, alongside the investigation of their identity. In fact, the specimens studied do not key to any of the known European species of Mantodea. A combination of characters such as complete development of wings, simple cerci, no foliaceous leg extensions, small size and an elongated pronotum (in contrast to all the *Ameles* spp.) among others place the available specimens in the genus *Miomantis*.

*Miomantis* Saussure, 1870 currently includes 70 Afrotropical species (Ehrmann 2002) and had not yet been recorded in Europe. The only available key for the genus is that of Giglio-Tos (1927) but this is largely based on morphological characters not necessarily relevant for phylogenetic species delimitation or are based on coloration and isometric scaling. *M. paykullii* Stal, 1871 is the most widespread species in the genus, almost ubiquitous south of the Sahara and present in Egypt and Mauritania (Ehrmann 2002, Battiston et al. 2010). This is the species found in Portugal first although followed closely by a second observation of a conspecific individual suggesting that there is an established population. Almost a month after this finding, five other males were independently located 200km to the north of the first records. These key out to the same genus but the slightly different morphology places them in the closely related species, *Miomantis caffra* Saussure, 1871 which is found naturally in South Africa and as an alien invasive in New Zealand (Ramsay 1984, Ramsay 1990). Both species, the genus *Miomantis* and tribe Miomantini are faunistic novelites to Portugal and the European continent.

## Materials and methods

The first specimen of *M. paykullii* was collected by hand at a garden light around which it was flying. A second specimen was observed 3 days later in the same place by beating the vegetation (*Arbutus unedo* and *Phyllostachys aurea* bamboo hedge) but flew off to an inaccessible area. The third and fifth to seventh specimens, belonging to *M. caffra* were attracted to a porch light during the night and intercepted hunting insects attracted by it. The fourth specimen, also *M. caffra*, was located in a different locality. All six collected specimens of *Miomantis* spp. were maintained alive till further analysis. Later, these were prepared and mounted dry, remaining in the author's personal reference collection. The captured specimen of *M. paykullii* and the first of *M. caffra* were photographed by the author.

## Taxon treatments

### Miomantis paykullii

Stal, 1871

#### Materials

- **Type status:** Other material. **Occurrence:** recordedBy: Eduardo Marabuto; individualCount: 1; sex: male; **Taxon:** genus: Miomantis; specificEpithet: paykullii; taxonRank: species; scientificNameAuthorship: Stal, 1871; vernacularName: Egyptian pygmy mantis; **Location:** continent: Europe; country: Portugal; countryCode: PT; stateProvince: Faro; county: Loulé; municipality: Quarteira; locality: Vila Sol; verbatimElevation: 40; decimalLatitude: 37.090; decimalLongitude: -08.093; **Event:** samplingProtocol: ad hoc observation; eventDate: 2014-08-05; habitat: garden
- **Type status:** Other material. **Occurrence:** recordedBy: Eduardo Marabuto; individualCount: 1; sex: male; **Taxon:** genus: Miomantis; specificEpithet: paykullii; taxonRank: species; scientificNameAuthorship: Stal, 1871; vernacularName: Egyptian pygmy mantis; **Location:** continent: Europe; country: Portugal; countryCode: PT; stateProvince: Faro; county: Loulé; municipality: Quarteira; locality: Vila Sol; verbatimElevation: 40; decimalLatitude: 37.090; decimalLongitude: -08.093; **Event:** samplingProtocol: vegetation beating; eventDate: 2014-08-08; habitat: garden

#### Description

Adapted from Giglio-Tos 1927, Ehrmann 2002 and Agabiti et al. 2010: Small or medium sized species. Green or light-brown coloured, patternless except occasional obscured humeral vein. Head broader than the pronotum, especially in the male. Eye prominent, slightly conical, more apparent in male but not ending in a spine. Pronotum slender with weakly developed supracoxal dilation, as long as or longer than fore coxa and smooth in male, finely toothed in female. Forewing hyaline in male, more opaque in female, distally dilated and slightly exceeding the tip of the abdomen in the former, only reaching the base of the cerci in the latter. Male hindwing hyaline, female yellowish, crossed by yellow veins. Supra-anal plate longer than broad, triangular. Foreleg unpatterned, coxa finely toothed with 5-6 spaced small spines stronger in female. Femur with 4 discoidal spines and 4 external. Fore tibia with 7 external spines. Body length: 36-39mm; pronotum length: 11-12mm in male, to 14mm in female; forewing length 23mm in male, 19-21mm in female.

##### Portuguese specimens

Both observed specimens are adult-stage males conforming well with the descriptions of the species available (Ehrmann 2002, Battiston et al. 2010). The first is illustrated in Fig. 1. This specimen is a straw-coloured and patternless mantis with a darker forewing radial vein. Both forewings and hindwings are hyaline, forewings being slightly less transparent and brownish. Head is short with prominent conical eyes. The raptorial forelegs bear the typical spine scheme of the genus and species: 5-6, 4, 7. Biometrics: pronotum length: 10mm; whole body length: 38mm; forewing length: 26mm; fore-leg length measurements - coxa: 6.6mm, femur: 8.2mm, tibia: 4.7mm; ratio forewing/ pronotum length: 2.6.

#### Distribution

An Afrotropical species cited throughout the biogeographical area, with some island populations. Countries where it has been found are: Burkina Faso, Cameroon, Chad, Egypt, Ghana, Israel, Ivory Coast, Kenya, Mauritania, Mauritius, Mozambique, Niger, Senegal, Togo, Uganda and Zimbabwe (Ehrmann 2002, Battiston et al. 2010).

##### Recorded habitats and distribution in Portugal

The area is a private property garden of the resort Vila Sol where many exotic plant species are planted. Among them, hedges of *Phyllostachys aurea* and *Lantana camara* are typical while stands of *Pennisetum alopecuroides*, *Cyperus papyrus* and garden turf are widespread with smaller amounts of other exotic species. The only natives are isolated *Pinus pinea* trees, remnants from the open woodland pre-resort and a hedge of *Arbutus unedo*. At dusk, several garden lights at ground level are regularly lit and during the night, an automatic irrigation system maintains humidity levels high, even during the summer. Prior to the installment of the resort, the whole area was a dry thermomediterranean open *Pinus pinea* woodland with mild winters and warm summers on an arenite substrate. The biogeographic province is the Gaditano-Onubo-Algarviense in its Algarviense coastal sector with some notable endemics (Rivas-Martinez et al. 1990, Costa et al. 1999) and seriously under threat from urban development. This particular site is one already lost as a natural habitat and is home to only the most resilient and human-adapted species.

#### Ecology

Ecological plasticity in this species over a broad temperature range accounts for its wide distribution. According to Prete et al. (1999), in Ghana *M. paykullii* mostly inhabits grasslands, the colour of which (a proxy for humidity levels) determines the final coloration and proportion between brown and green morphs. Presumably, a brown morph develops in a less moist environment. In Ghana, adults display no deimatic behaviour and attempt to fly or walk away from danger (Edmunds 1972). Adults are also presumably very mobile and active at night and especially sensitive to bat echolocation high frequency sounds (80-100 KHz), thus being able to evade predation (Prete et al. 1999). This phenomenon has been experimentally tested with the closely related *M. natalica* Beier, 1930 by Cumming (1996).

#### Conservation

Showing a wide distribution centred in the subsaharan African continent, *M. paykullii* should not be at risk of any kind. However, in the Euro-Mediterranean area, where it has only been found along the Nile valley and nearby areas of Israel, this species has recently been evaluated as at "Potential risk", because of sparse observations for a long time (Agabiti et al. 2010).

### Miomantis caffra

Saussure, 1871

#### Materials

- **Type status:** Other material. **Occurrence:** recordedBy: Eduardo Marabuto; individualCount: 1; sex: male; **Taxon:** genus: Miomantis; specificEpithet: caffra; taxonRank: species; scientificNameAuthorship: Saussure, 1871; **Location:** continent: Europe; country: Portugal; countryCode: PT; stateProvince: Lisboa; county: Cascais; municipality: Carcavelos; locality: São Miguel das Encostas; verbatimElevation: 50; decimalLatitude: 38.701; decimalLongitude: -9.336; **Event:** samplingProtocol: ad hoc observation; eventDate: 2014-09-09; habitat: garden
- **Type status:** Other material. **Occurrence:** recordedBy: Eduardo Marabuto; individualCount: 1; sex: male; **Taxon:** genus: Miomantis; specificEpithet: caffra; taxonRank: species; scientificNameAuthorship: Saussure, 1871; **Location:** continent: Europe; country: Portugal; countryCode: PT; stateProvince: Estremadura; county: Lisboa; municipality: Oeiras; locality: Quinta do Marquês; verbatimElevation: 35; decimalLatitude: 38.696; decimalLongitude: -9.329; **Event:** samplingProtocol: ad hoc observation; eventDate: 2014-09-20; habitat: garden
- **Type status:** Other material. **Occurrence:** recordedBy: Eduardo Marabuto; individualCount: 2; sex: male; **Taxon:** genus: Miomantis; specificEpithet: caffra; taxonRank: species; scientificNameAuthorship: Saussure, 1871; **Location:** continent: Europe; country: Portugal; countryCode: PT; stateProvince: Lisboa; county: Cascais; municipality: Carcavelos; locality: São Miguel das Encostas; verbatimElevation: 50; decimalLatitude: 38.701; decimalLongitude: -9.336; **Event:** samplingProtocol: ad hoc observation; eventDate: 2014-09-22; habitat: garden
- **Type status:** Other material. **Occurrence:** recordedBy: Eduardo Marabuto; individualCount: 1; sex: male; **Taxon:** genus: Miomantis; specificEpithet: caffra; taxonRank: species; scientificNameAuthorship: Saussure, 1871; **Location:** continent: Europe; country: Portugal; countryCode: PT; stateProvince: Lisboa; county: Cascais; municipality: Carcavelos; locality: São Miguel das Encostas; verbatimElevation: 50; decimalLatitude: 38.701; decimalLongitude: -9.336; **Event:** samplingProtocol: ad hoc observation; eventDate: 2014-09-27; habitat: garden

#### Description

Adapted from Giglio-Tos (1927), who placed it as a synonym of *M. monacha* (Fabricius, 1787) and Ramsay (1990). Small or medium-sized species. Male green-coloured (occasionally brown) but distal part of pronotum may be obscured reddish brown. Wing hyaline except green area along space between radial and medial veins and pale, often contrasting radial vein. Female larger, stouter, pastel green with opaque green forewing and yellow hindwing. Head broader than the pronotum, especially in the male. Eye prominent globular bulging, more apparent in male. Pronotum slender, as long as or longer than fore coxa and smooth in male, finely toothed in female. Forewing distally dilated and exceeding the tip of the abdomen and cerci in male, much shorter not reaching the tip of abdomen in female. Foreleg patterned with inner coxa bearing 4-6 dark spots and finely toothed with 5-6 spaced small spines, stronger in female. Femur with 2-3 dark spots interiorly, 4 discoidal and 4 external spines. Fore tibia with 7 external spines. body length: 40-43mm; pronotum length: 10-13mm in male, to 16mm in female; forewing length 31mm in male, 22mm in female.

##### Portuguese specimens

The six observed and collected individuals of *M. caffra* are adult specimens found by porch lights at night. All are identical in pattern and therefore only the first is described, while measurements are also given for the remainder. It is a match for the description in Giglio-Tos 1927 but particularly Ramsay 1990 and is represented in Fig. 2. This specimen is bright green with metazone (distal section of pronotum) and femora with a pale reddish brown suffusion. Forewings are hyaline except the green radial-medial and a pronounced white medial vein. Head bears large bulging and round eyes and does not appear depressed as *M. paykullii*. Abdomen upperside is bright yellow. Raptorial forelegs bear the typical spine scheme and pattern of the genus and species: 5-6, 4, 7 with 4 dark spots on coxae and 3 prominent black femoral spots and darkened spines. Biometrics, n=6: pronotum length: 11 (10-11)mm; whole body length: 37 (36-38) mm; forewing length: 29 (27-31) mm; fore-leg length measurements - coxa: 7 (6-7)mm, femur: 9 (8-9)mm, tibia: 5 (4-5)mm; ratio forewing/ pronotum length: 2.7 (2.7-2.8).

#### Distribution

Originally endemic to the extreme south of Africa in South Africa and Mozambique. Original area spans from Cape of Good Hope to Maputo Bay, the former Transvaal province and Natal (Giglio-Tos 1927). Now, *M. caffra* is an alien and spreading species in New Zealand's North Island around Auckland (Ramsay 1984, Ramsay 1990).

##### Recorded habitats and distribution in Portugal

The area surrounding sightings of *M. caffra* is a suburban neighbourhood where managed gardens are abundant. *Paspalum* spp. lawns are widespread interspersed with stands of *Lantana camara*, *Pittosporum tobira*, *Yucca aloifolia*, *Nerium oleander* and cultivated *Rosa* spp. Other widely planted exotic species include *Buxus sempervirens*, *Ligustrum ovalifolium*, *Phoenix canariensis* and *Hibiscus* spp. Such gardens are watered every night and the whole environment contrasts with natural vegetation types. Here, the natural series would be a meso-thermomediterranean xerothermophilous vegetation adapted to a limestone substrates, the woodland series *Arisaro clusii- Querco broteroi sigmetum*, typical of western Portugal (Mesquita et al. 2005). Climax stands of this vegetation series are scarce and the more open and thermic seral stages occupy the now few but biodiverse fragmented areas.

#### Ecology

All from Ramsay (1990). Overall similar to *M. paykullii*. Presumably a generalist and adaptive species with a preference for warm-temperate situations. *M. caffra* is annual and females live longer than males as most of the latter are eaten during copulation. Development time is variable and nymphs do not synchronise emergence, reaching adult stage at different times, not necessarily depending on temperature. While males are capable of oriented flight and are attracted to lights, females only glide, at best.

## Checklists

### Updated checklist of the Mantodea in Portugal

#### Amorphoscelidae

#### Perlamantis allibertii

Guerin-Méneville, 1843

##### Distribution

In Portugal this species is known from a wide inland area throughout the whole country.

##### Notes

Seabra (1937), Grosso-Silva and Soares-Vieira (2004), Marabuto et al. (2014)

#### Empusidae

#### Empusa pennata

(Thunberg, 1815)

##### Distribution

A widespread species in Portugal, found throughout the country.

##### Notes

Bolívar 1876

#### Mantidae

#### Ameles spallanzania

(Rossi, 1792)

##### Distribution

In Portugal a regular and widespread species.

##### Notes

Battiston et al. (2010), Agabiti et al. (2010)

#### Ameles paradecolor

Agabiti, Salvatrice & Lombardo, 2010

##### Distribution

Throughout the whole of Portugal but uncommon, favouring Mediterranean inland areas.

##### Notes

Agabiti et al. 2010

#### Apteromantis aptera

(Fuente, 1894)

##### Distribution

Only locally common species, limited to the southern half of Portugal in Mesomediterranean open areas.

##### Notes

Grosso-Silva and Soares-Vieira (2004), Boieiro et al. (2007), Marabuto et al. (2014)

#### Geomantis larvoides

Pantel, 1896

##### Distribution

In Portugal, throughout the country but preferring sandy areas near the coast.

##### Notes

Battiston et al. 2010

#### Mantis religiosa

(Linnaeus, 1758)

##### Distribution

Common and throughout the whole of Portugal in both natural and urban areas.

##### Notes

Battiston et al. 2010

#### Miomantis paykullii

Stal, 1871

##### Distribution

Extreme south of Portugal in the Algarve under subtropical semi-natural conditions. Only in the Quarteira area (Loulé, Faro, Algarve) based on two specimens.

##### Notes

This work

#### Miomantis caffra

Saussure, 1871

##### Distribution

Suburban area of Lisboa, Portugal in anthropised semi-natural conditions. Only in the area of Carcavelos and Oeiras (Cascais, Lisboa), based on five male specimens.

##### Notes

This work.

#### Sphodromantis viridis

(Forskal, 1775)

##### Distribution

Only known from inland areas near the border with Spain in the southern half of Portugal.

##### Notes

Marabuto et al. 2014

#### Tarachodidae

#### Iris oratoria

(Linnaeus, 1758)

##### Distribution

Throughout Portugal but more abundant at the end of summer and in the Mediterranean areas of the south.

##### Notes

Bolívar (1876)

## Identification Keys

### Key to Portuguese species of Mantodea

**Table:** 

1	Wings absent	2
–	Wings present	3
2	Eyes conical or pointed. Green to yellowish brown coloration.	*Apteromantis aptera* (Fuente, 1894)
–	Eyes globular, round shape. Body dark grey to brown, mottled and abdomen bearing a dorsal stripe.	*Geomantis larvoides* Pantel, 1896
3	Pronotum short, <2x or = length of head	*Perlamantis allibertii* Guérin-Méneville, 1843
–	Pronotum longer than 2x length of head	4
4	Head with a conical process, mid and hind-femora and abdomen underside with tubercles or foliose projections	*Empusa pennata* (Thunberg, 1815)
–	Head and body without such structures	5
5	Forewing long, reaching the tip of the abdomen, with a central white or cream-coloured spot (stigma)	*Sphodromantis viridis* (Forskal, 1775)
–	Forewing long or short, without white or cream area	6
6	Hindwing with a large dark coloured, blue sheen ocellus in the anal field. This is often surrounded by red, yellow or orange areas. Wings to tip of abdomen in male, shorter in female.	*Iris oratoria* (Linnaeus, 1758)
–	Hindwings without ocellus, monochromatic. If not transparent, yellow, black or red.	7
7	Dark spot on internal fore-coxa, often centred yellow or white and exposed during deimatic display	*Mantis religiosa* (Linnaeus, 1758)
–	Without dark spot in the fore coxa	8
8	Body length > 4x pronotum length. Stouter species with thicker pronotum and raptorial forelegs. Wings fully developed in male, very short in female.	Genus *Ameles*, 9
–	Body length < 4x pronotum length. Slender species with prominent eyes. Fully winged, in male reaching or passing tip of abdomen, in female shorter.	10
9	Ratio pronotum length / maximum width <2. Eyes ovoid with an apical spine.	*Ameles spallanzania* (Rossi, 1792)
–	Ratio pronotum length / maximum width >2. Eyes rounded and not prominent.	*Ameles paradecolor* Agabiti, Salvatrice & Lombardo, 2010
10	Eyes conical and head appearing compressed dorsally. Wings hyaline in male, exceeding only slightly the tip of abdomen, but not cerci. In female, slightly shorter than abdomen. Abdomen uniform colour dorsally and ventrally. Unpatterned frontal raptorial legs.	*Miomantis paykullii* Stal, 1871
–	Eyes globular. Wings hyaline in male exceeding tip of cerci. In female, much shorter than tip of abdomen. Abdomen dichromatic, yellow dorsally. Raptorial forelegs with small black dots especially in femora.	*Miomantis caffra* Saussure, 1871

This key is to be used with specimens in the adult stage. In nymphs, the absence of developed wings may lead to erroneous identifications.

## Analysis

The first Portuguese and European specimens of wild-caught mantises in the genus *Miomantis* were observed in late Summer 2014.

**Figure 3.**
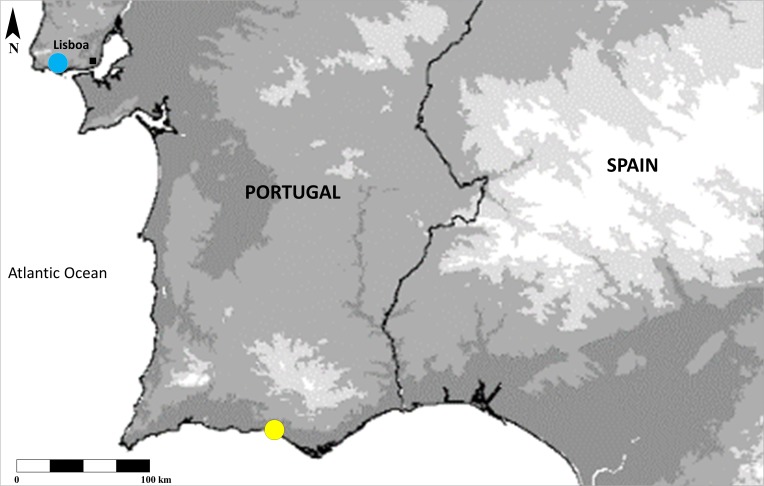
Distribution map of the *Miomantis* spp. citations in this work. *Miomantis paykullii* - yellow circle. *Miomantis caffra* - blue circle.

*Miomantis paykullii*, was located in Vila Sol, Quarteira, Algarve, Portugal from two observations separated by three days in August. The second specimen escaped, preventing further inspection other than sexing and identification. One month later, six males of *Miomantis caffra* were collected in the area of Carcavelos/Oeiras, Lisboa, Portugal at fluorescent porch lights to which moths, lacewings and beetles (potential prey) are regularly attracted. Known distribution of both species in Portugal is now represented in Fig. 3.

To accomodate these findings, the 11 species of Mantodea in Portugal are revised giving the general distribution of each species in the country and citing relevant literature. With the aim of supporting further studies and aiding recording, a key for the identification of adult specimens of all species within the country (view Identification key) is included.

## Discussion

With the discovery of both *Miomantis paykullii* and *Miomantis caffra* in Portugal during the summer of 2014, there are now 11 mantis species known in the country and 39 in the European continent. The biogeographical realm of the genus *Miomantis* is the Afrotropical region, at least 2000 km away from Portugal and where all the 70 known species are endemic.

The straightforward assignment of specimens to known and relatively widespread taxa and their discovery in heavily modified habitats in European territory probably indicate faunistic novelty rather than natural relictual occurrence. *M. paykullii* and *M. caffra* are therefore, the first alien mantises in the European continent and are among the relatively few other cases known in the world (see introduction).

The Euro-Mediterranean biome is a renowned hotspot for biological diversity (Myers et al. 2000) and on a crossroad of different biogeographic realms. However, it is also a stressful environment and arguably resistant to invasion. Biota have to adapt to several constraints including periodic drought and irregular rains, high seasonal temperatures, forest-fires, competition from other species, and a great long-standing human pressure. Nevertheless, it is currently one of the regions most affected by alien species (di Castri et al. 1990, Groves and Di Castri 1991) and their threats: from habitat displacement to active depredation and competition.

These findings enhance and are derived from the increasing pressure biodiversity is facing with rising worldwide biotic homogenisation (McKinney and Lockwood 1999). With this phenomenon, some species-traits benefit from loosened biogeographic barriers and human action on otherwise difficult to disrupt ecosystems. Current evidence is insufficient to show that these findings represent a long-term colonisation of Europe by two African species, but the ecological plasticity of both and the historical ability of *M. caffra* to establish in an alien ecosystem is indicative that it is already taking place. Further, the geographical distance between the two discoveries, involving two different species reinforces the idea of independent introductions. As to their provenance, factual data is lacking but some hypotheses can be put forward.

All Portuguese *Miomantis* spp. were collected in completely man-engineered habitats buffered from Mediterranean habitats. In these gardens, commercial and widespread garden plants are used and watering is regular, establishing a subtropical environment and hampering many native species to settle, compete or interfere.

The first hypothesis to their origin includes long range natural dispersal from original distribution areas. This would involve a range expansion of from 2000 to 4000 km by *M. paykullii* from either Mauritania or Egypt. In this case, man-made habitats like gardens and parks would act as stepping stones overcoming biogeographical barriers. This is even less likely for *M. caffra* which is endemic to South Africa. Even though gardens, parks and suitable areas are fairly continuous in Europe and maybe the Maghreb, there are still wide expanses of unsuitable (either too dry or sparsely populated) ground to allow these species to spread naturally. Moreover, reaching the Iberian Peninsula as the leading edge of a widening distribution area would indicate a presence in countries in-between, which has not yet been verified.

In the second hypothesis, worldwide trade allows for a fast and intense exchange of goods, easily overcoming natural biogeographic boundaries. All developmental stages but especially oothecae attached to solid surfaces and nymphs on potted plants would be easily transportable. This has happened before, as in the case of the Japanese mantis *Statilia maculata* reaching New Zealand (Harris 2007). The prevalence of exotic species and the great turnover of people where *M. paykullii* was found (a popular holiday resort for people coming from all over Europe) make this a likely explanation for its arrival, although asking gardeners about recently imported Afrotropical goods resulted in no positive results. The area where *M. caffra* was located is also prone to the effects of global trade because of its short distance to the major ports of Lisboa and Cascais. Finally, both *M. paykullii* and *M. caffra* are widely available in the pet trade (particularly on the Internet). Both species are bred for themselves or used as live reptile food and their acquisition is straightforward and bound by no regulations within the European Union. Whether these findings result from accidental introductions or escapees is still unknown.

Beside the potential threat an alien predator such as *Miomantis* spp. may impose on potential prey species (i. e. all smaller arthropods), these mantises could also compete with autochthonous species. In Portugal, the only species that venture regularly into gardens and parks are *Mantis religiosa* and to a much lesser extent *Iris oratoria* and *Empusa pennata*. These species attain on average a larger size and are more strongly built than *Miomantis* spp., meaning that direct predation by the alien upon them is unlikely. However, *Miomantis* species are aggressive generalists which might prey on the same insects and thus out-compete natives. *M. caffra* itself has proven to be displacing *Orthodera novaezelandiae*, the only New Zealand native mantis in the outskirts of Auckland (Ramsay 1990). Furthermore, the introduced *M. caffra* in New Zealand has proven its interaction with *O. novaezelandiae* goes beyond pure competition. It has been shown that by sexual deceit, *M. caffra* females will attract males of the native species willing to mate (Fea et al. 2013). The latter are then eaten and mating is not accomplished. It is not known if the interaction with European native species renders the same outcome through pheromone overlapping but unlike *O. novaezelandiae*, sexual cannibalism is a widespread behaviour in European mantises (Battiston et al. 2010).

Whether these *Miomantis* populations are already established or are just the result of episodic releases or escapees, the potential disturbance to the ecosystem requires immediate monitoring and research. The extent of their influence is for now unknown as well as the means of their arrival and therefore a call for action is required.

### Conclusions

As the Mediterranean depletion of biodiversity increases due to urban pressure, forest fires and engulfment in invasive alien species (e.g. Rundel et al. 1998), climate change is expected to have the greatest impacts on biodiversity (Sala et al. 2000, Walther et al. 2009). With biotic homogenisation, even such a biodiverse and resilient system as the Mediterranean biome is impacted and the consequences are relevant (Olden et al. 2004) especially when triggered by invasions (Mooney and Cleland 2001, Clavero and García-Berthou 2005). Moreover, in spite of important projects like DAISIE (http://www.europe-aliens.org/), early monitoring of alien species in the European continent is mostly restricted to the initiative of keen naturalists and scientists currently working on other subjects. This is especially true in the current Portuguese context where science research has recently suffered important budget cuts (Levy 2014) and government funding is scant for the subject of invasive species. Portugal is definitely a gateway to Europe in many aspects, because of its geographical position, mild climate, relaxed biosafety measures but also because the alarm system lacks definition. Once in European territory, the problem of invasive species is no longer only a national concern because of European free market and easy movement of people and goods. Natural and ecological barriers to dispersal are reduced and whether it is a pet, a nymph or larva in a potted plant or a hitchhiking ootheca naturally glued to a car surface there is an ever increasing chance it becomes naturalised, with economical, social, aesthetic and life-threatening impacts.

## Supplementary Material

XML Treatment for Miomantis paykullii

XML Treatment for Miomantis caffra

XML Treatment for Amorphoscelidae

XML Treatment for Perlamantis allibertii

XML Treatment for Empusidae

XML Treatment for Empusa pennata

XML Treatment for Mantidae

XML Treatment for Ameles spallanzania

XML Treatment for Ameles paradecolor

XML Treatment for Apteromantis aptera

XML Treatment for Geomantis larvoides

XML Treatment for Mantis religiosa

XML Treatment for Miomantis paykullii

XML Treatment for Miomantis caffra

XML Treatment for Sphodromantis viridis

XML Treatment for Tarachodidae

XML Treatment for Iris oratoria

## Figures and Tables

**Figure 1a.**
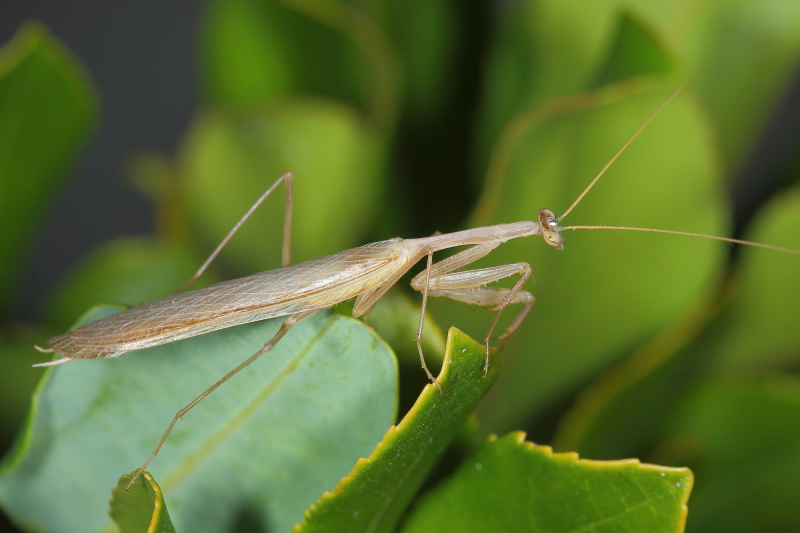
Dorso-lateral habitus view. Note hyaline forewings with obscured radial vein.

**Figure 1b.**
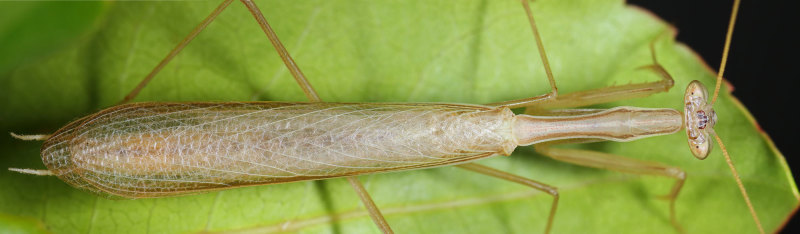
Dorsal view. Note the smooth lateral side of relatively long pronotum, straight frons, simple cerci and hyaline wings.

**Figure 1c.**
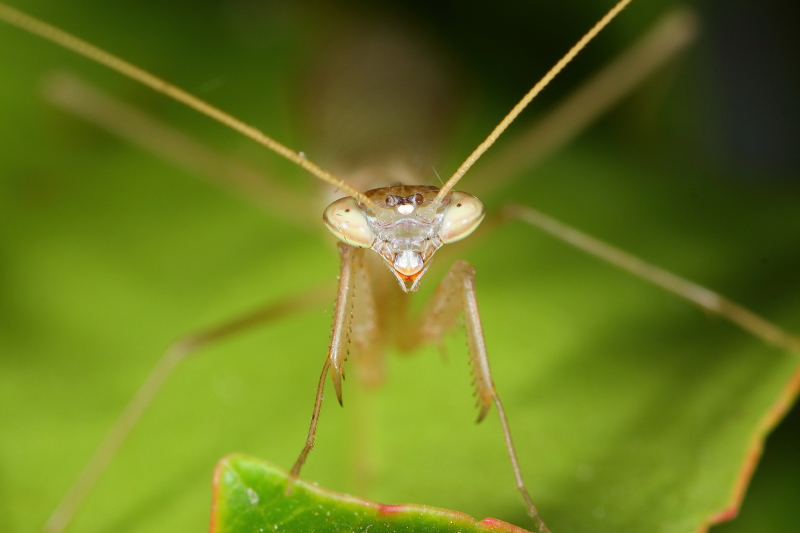
Head frontal view. Note the conical, prominent and striped eyes.

**Figure 1d.**
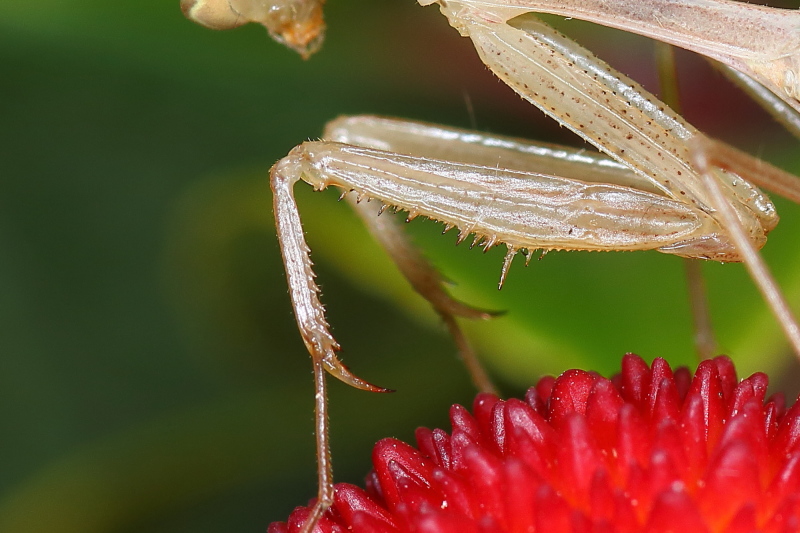
Detail of foreleg. Characteristic are the coxa bearing very small spines (5-6), femora crenulate between the 4 external spines and tibiae with 7 external spines.

**Figure 2a.**
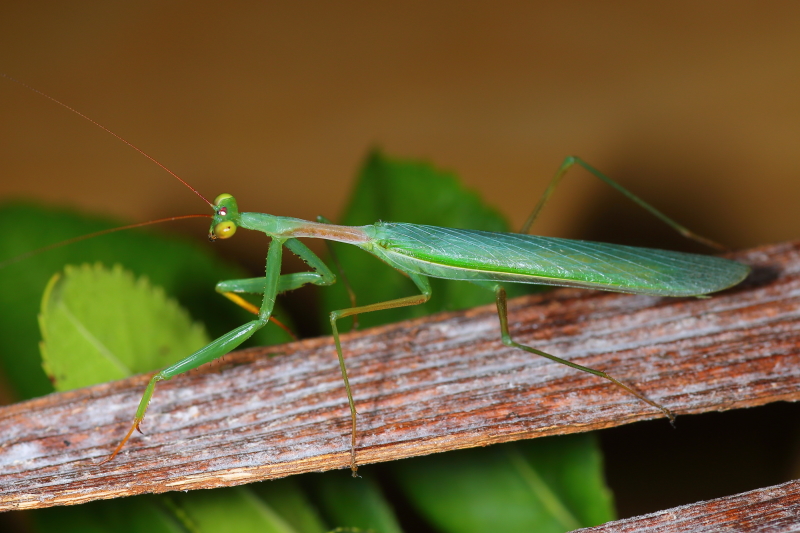
Dorso-lateral habitus view. Note obscured metazone and femora on otherwise green colouration and white radial vein of forewings.

**Figure 2b.**
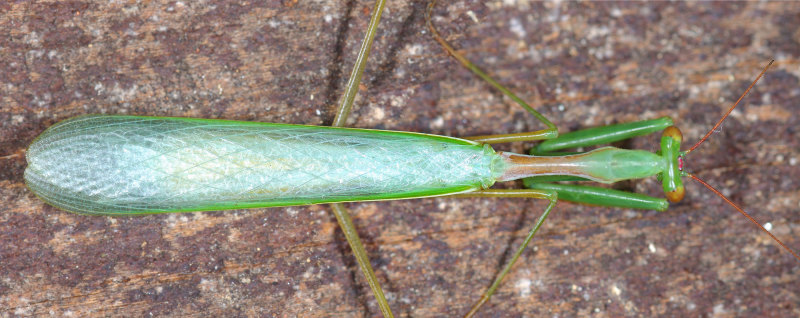
Dorsal view. Note the smooth lateral side of relatively long pronotum, bulging globular eyes, longer wings than abdomen extending beyond cerci.

**Figure 2c.**
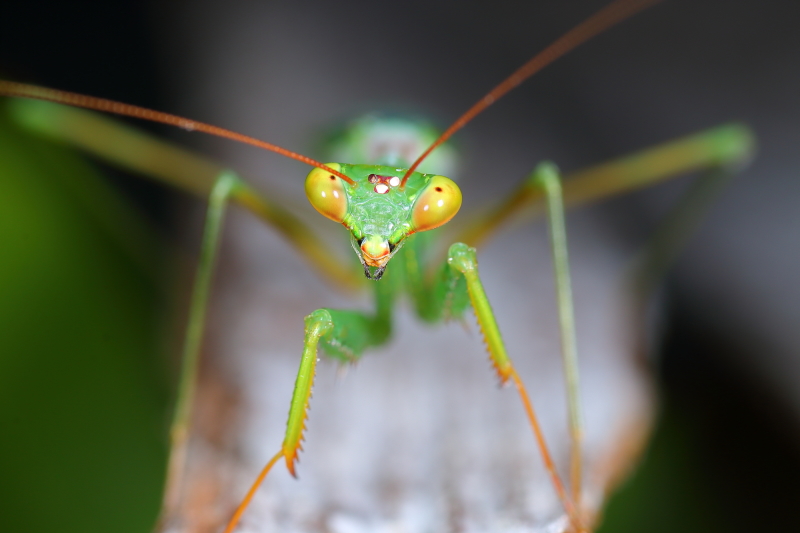
Head, frontal view. Note the round, bulging eyes.

**Figure 2d.**
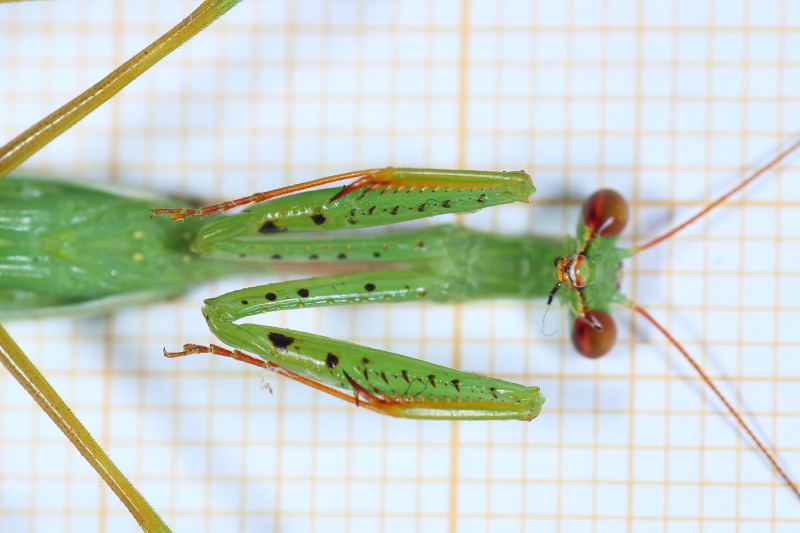
Detail of inner part of forelegs. Characteristic are slightly spotted coxae (5-6 black patches) and femora (3 patches).
